# Studies on the Mechanical Models and Behaviors for the Stamp/Film Interface in Microtransfer Printing

**DOI:** 10.3390/ma15175915

**Published:** 2022-08-26

**Authors:** Mengjie Wu, Yuyan Zhang, Xin Dai, Ling Jiang

**Affiliations:** College of Mechanical and Electronic Engineering, Nanjing Forestry University, Nanjing 210037, China

**Keywords:** stamp/film interface, adhesion/delamination characteristics, J-integral, virtual crack closure technology, cohesive zone model

## Abstract

The adhesion/delamination characteristics at the stamp/film interface are critical for the efficiency of film microtransfer printing technology. To predict and regulate the interface mechanical behaviors, finite element models based on the J-integral, Virtual Crack Closure Technology (VCCT), and the cohesive zone method (CZM) were established and compared. Then, the effects of pulling speed and interface parameters on the pull-off force, which is used to characterize the interface adhesion strength, were investigated. Comparisons between the simulation results and previous experimental results demonstrated that the model based on the CZM was more applicable than the models based on the J-integral and VCCT in analyzing the adhesion/delamination behaviors of the stamp/film interface. Furthermore, the increase in pulling speed could enlarge the pull-off force for the viscoelastic stamp/film interface, while it had no influence on the pull-off force for the elastic stamp/film interface. In addition, a larger normal strength and normal fracture energy resulted in a larger pull-off force, which was beneficial to the realization of the picking-up process in microtransfer printing.

## 1. Introduction

Flexible inorganic electronic devices, which are prepared by integrating ultrathin functional elements based on traditional inorganic semiconductor materials with flexible substrates, have shown attractive application prospects in the fields of the optical/electronic industry and medical treatment due to their good electronic properties and unique flexibility [1]. One key step in the fabrication of flexible inorganic electronic devices is the epitaxial growth of inorganic thin films [2]. To meet this processing requirement, multiple thin film transfer techniques have been developed, such as temporary wafer bonding [3] and transfer printing methods [4]. Among these techniques, microtransfer printing is one of the most widely used transfer printing methods due to the advantages of environment-independent temperature/corrosion properties, repeatability, and high precision [5]. The principle of the microtransfer printing technique is to transfer thin film components prefabricated on donor substrate (i.e., inorganic semiconductor substrate) to target substrate (i.e., flexible substrate) using a flexible stamp. Two steps including picking-up and printing are commonly involved in this technique. In the picking-up step, the stamp is brought into contact with the thin film and then peeled away to remove the film from the donor substrate, while in the printing step, the inked stamp contacts the target substrate and is retrieved to print the film onto the target substrate. The interfacial adhesion caused by van der Waals interactions at the micro scale is the pivotal factor influencing these two steps [5]. Successful microtransfer printing requires that the adhesion at the stamp/film interface is stronger than that at the film/donor substrate interface to delaminate the film and the donor substrate in the picking-up step, while it is weaker than that at the film/target substrate interface to delaminate the film and the stamp in the printing step [6]. However, the above requirements cannot always be satisfied, and controls of adhesion and delamination at interfaces, especially the stamp/film interface, still have difficulties [7,8]. To date, obtaining a high fabrication yield remains challenging [5,9,10], and thus the industrial application of microtransfer printing is delayed [1]. To optimize the existing microtransfer printing technologies and develop new efficient methods, many efforts have been made to develop methods for regulating the adhesion between stamp and film [1,5]. Evaluating the feasibility of adhesion regulation methods primarily requires prediction of the mechanical behaviors of the stamp/film interface.

As far as the numerical techniques for predicting the adhesion/delamination characteristics of the stamp/film interface are concerned, three kinds of mechanical models including a model based on J-integral theory, a model based on the virtual crack closure technique (VCCT), and a model based on the cohesive zone method (CZM) are mainly used. Both the model based on J-integral theory and that based on the VCCT treat the interface adhesion/delamination in the microtransfer printing process as a crack propagation problem and evaluate the adhesion or delamination state of the interface according to the energy release rate, defined as the energy per area necessary to cause the crack to propagate [7,11]. The J-integral is an energy contour integral proposed to quantitatively characterize the strength of the stress and strain field around a crack tip, which equals the energy release rate for linear elastic fracture problems [12]. Based on the J-integral method, Tucker et al. [7] established a mechanical model of the stamp-film-substrate system and calculated the energy release rate by introducing initial cracks at the interface and using finite element simulation. Effects of the initial crack length and interface toughness on the interface adhesion/delamination behaviors were investigated. However, the stamp was modeled as an elastic solid. Cheng et al. [13] abandoned the elastic assumption of the stamp and proposed a viscoelastic model for calculating the energy release rate at the stamp/film interface from the J-integral. The pull-off force required to fail the interface was obtained by setting the energy release rate equal to the interface toughness, and the superiority of the finite element model to the analytical model in revealing the relationship between pull-off force and pulling speed was demonstrated. Similar to the J-integral method, the VCCT method, which was proposed based on the Irwin energy theory [14], is another commonly used technique to calculate the energy release rate. This method assumes that the strain energy released upon an increment of crack growth is equal to the energy required upon the same increment of crack closure. Based on the VCCT method, Carlson et al. [15] illustrated influences of the shear displacement of the stamp on the energy release rate by establishing and solving a finite element model of the stamp–film system containing an interfacial preset crack. Simulation results of the pull-off force reflected similar trends to the experimental ones even though the preset crack length was arbitrary. Furthermore, the VCCT method was also adopted by Kim-Lee et al. [11] and Minsky et al. [16] to examine the effects of different factors on the adhesion behavior at the stamp/film interface and provide understanding of the mechanics of interface delamination. However, in the above three studies, the material of the stamp was assumed to be linear elastic. Different from the J-integral method and VCCT method, the model based on the CZM treats the interface adhesion/delamination in the microtransfer printing process as an interface damage problem and evaluates the adhesion or delamination state of the interface according to the cohesive law [17]. In this method, a cohesive interface is defined at the stamp/film interface and the cohesive law characterizing the interface traction force–separation displacement relationship is used to describe the non-linear nature of the interface strength. Using the CZM approach, Jiang et al. [17] modeled the viscoelastic stamp/film interface and investigated the effects of the viscoelastic modulus and relaxation time of the stamp on the area of retrieved film in the stamp through finite element simulations. Subsequently, Al-okaily et al. [18] adopted the CZM approach to model the stamp/film interface thermo-mechanical delamination in the laser microtransfer printing technique and demonstrated the capabilities of this approach. Both of the above studies applied the bilinear cohesive law to represent the degradation and failure of the stamp/film interface.

Although the above mechanical models provide methods for predicting the interface adhesion/delamination behavior, their applicability is not clear as they are used separately in different studies. In order to determine the suitability of these three numerical calculation models, this paper intends to compare the results of these models and determine the most proper one. To achieve this, the layout of this paper is arranged as follows. First, interface mechanical models based on the J-integral theory, VCCT, and CZM are established for the microtransfer printing problem of a thin film by a flat stamp using the classical kinetically controlled operation mode. Then, these three models are solved separately by adopting the commercial finite element package Abaqus to obtain the adhesion/delamination behavior of the stamp/film interface. Their results are compared and analyzed to assess the applicability of different models and determine the most suitable model. Finally, the influences of the microtransfer printing technological parameters and material interface parameters on the mechanical behavior of the stamp/film interface are investigated. For the convenience of analysis, the materials of the film and stamp are selected to be silicon and polydimethylsiloxane (PDMS), respectively, which were adopted in most of the previous studies [7,11,15,16,17,18]. For other materials, the simulation approaches and calculation methods presented in this paper can also be applied.

## 2. Stamp/Film Interface Mechanical Models

**Figure 1:**
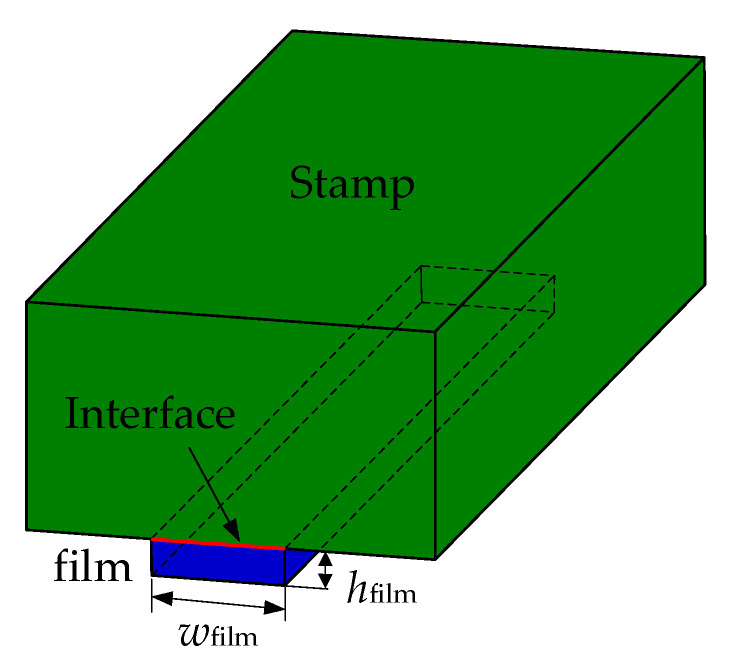
Schematic showing the stamp, film, and interface.

Figure 1 shows the schematic diagram of the stamp, film, and interface, in which the film thickness *h*_film_ is much smaller than the stamp thickness and the film width *w*_film_ is much smaller than its length. Therefore, the film and stamp can be taken to deform under the plane strain conditions. To predict the adhesion/delamination mechanical behaviors of the stamp/film interface, the models based on J-integral theory, the VCCT, and the CZM are established in this section and an energy-based criterion for crack propagation is used. The Griffith criterion [19] is a simple and effective fracture criterion selected in J-integral theory and VCCT [7,11].The crack propagation condition given by the Griffith criterion is *G* > *Γ*_0_, in which *G* denotes the energy release rate and *Γ*_0_ is the interface toughness measured by experiments. In J-integral theory, the J-integral value is equivalent to the energy release rate for linear elastic materials, which were assumed in previous studies [7,11]. The VCCT is a method proposed based on the Griffith criterion [20]. Furthermore, in the cohesive zone model, an energy-based crack propagation criterion in which the area enclosed under the traction–separation curve equals the critical energy release rate is often utilized [17]. Therefore, through the parameter energy release rate, the models based on the J-integral, VCCT, and CZM can be linked and compared. Brief introductions of the establishing methods for these three models are presented as follows.

### 2.1. Mechanical Model Based on J-Integral Theory for the Stamp/Film Interface

The J-integral denotes the path-independent contour integral around the crack tip [12], which is proposed based on the energy conservation principle and can evaluate the available energy to delaminate the given interface. Under the linear elastic fracture mechanics assumption, the J-integral value equals the energy release rate, and its definition for two-dimensional problems can be expressed as [12]

(1) $$J=\int_{\tau }\left(wdy-T_{i}\frac{\partial u_{i}}{\partial x_{i}}ds\right)$$

where $w=\int_{0}^{\varepsilon_{ij}}\sigma_{ij}d\varepsilon_{ij}$ and $T_{i}=\sigma_{ij}n_{j}$. *u_i_* denotes the displacement vector, d*s* is the increment of length along the integral path, *τ* represents the path around the crack tip, *w* is the strain energy density, and *T_i_* denotes the stress component at any point along the integral path. *σ_ij_* and *ε_ij_* are the stress and strain tensors, respectively, and *n_j_* is the unit normal vector along the integral path.

**Figure 2:**
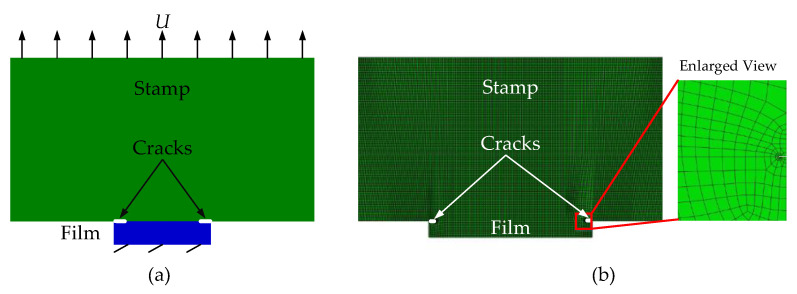
Schematics of the model based on J-integral theory for the stamp/film interface. (**a**) Mechanical model; (**b**) Finite element model.

In order to use the finite element method to calculate the J-integral at the crack tip of the stamp/film interface, cracks with length *c* at both ends of the interface need to be preset, as shown in Figure 2a. The two-dimensional finite element model established in Abaqus software [21] is shown in Figure 2b. The mesh elements near the crack tip are locally refined to improve the accuracy of calculation. Swept mesh is adopted in the refinement zone and generated along the sweep path to improve the mesh quality.

### 2.2. Mechanical Model Based on the VCCT for the Stamp/Film Interface

In the virtual crack closure technique (VCCT), the energy release rate is evaluated according to the force at the node of the crack tip and the displacement at the node behind the crack tip. For the two-dimensional problem, the energy release rate *G* for four-noded elements is calculated as [22]

(2) $$\left\{\begin{array}{c} G\approx G_{I}+G_{\mathrm{II}}\\ G_{I}=\frac{F_{y1}\Delta v_{3,4}}{2B\Delta a}\\ G_{\mathrm{II}}=\frac{F_{x1}\Delta v_{3,4}}{2B\Delta a} \end{array}\right.$$

where *G*_I_ and *G*_II_ are components of the energy release rate under crack-opening mode I and in-plane shear mode II, respectively. *B* is the thickness of the cracked body, Δ*a* is the micro-increment of crack, *F_x_*_1_ and *F_y_*_1_ are the force components acting on node 1 of crack tip, and Δ*v*_3,4_ is the opening displacement between nodes 3 and 4 behind crack tip.

**Figure 3:**
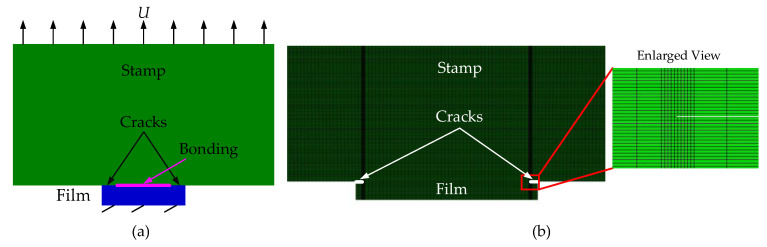
Schematics of the model based on the VCCT for the stamp/film interface. (**a**) Mechanical model; (**b**) Finite element model.

In order to calculate the energy release rate at the crack tip of stamp/film interface by combining the VCCT and finite element method, initial defects, i.e., cracks, should be specified at the interface. As shown in Figure 3a, the introduction of initial cracks with length *c* in the unbonded area at both ends of the interface can be realized by setting bonding units in the middle area of the interface. The two-dimensional finite element model established in Abaqus is shown in Figure 3b.

### 2.3. Mechanical Model Based on the CZM for the Stamp/Film Interface

**Figure 4:**
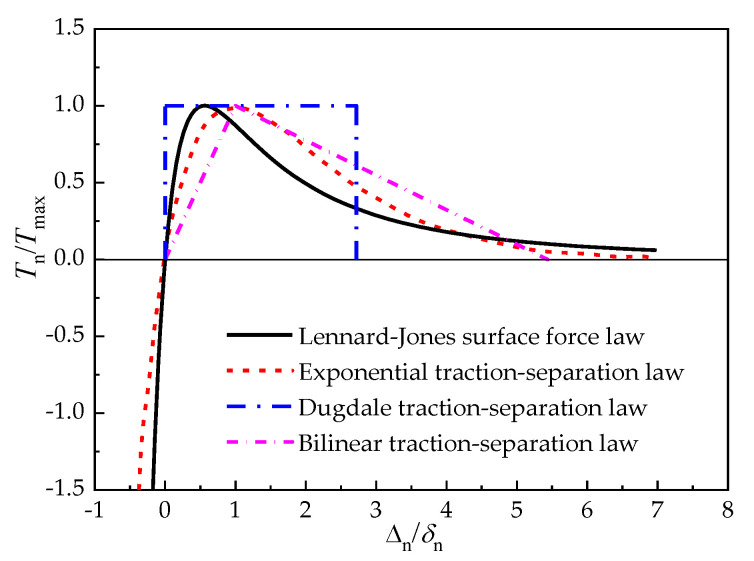
Different traction–separation laws under the crack opening mode.

The cohesive zone method (CZM) is a widely used theory based on damage mechanics for predicting crack initiation and propagation [23]. It regards the vicinity of the crack tip as a crack process zone [24], and the cohesive damage zone is formed by introducing the degradation mechanism (that is, the material softening or weakening) in front of the crack. The constitutive relationship between surface traction force and relative separation displacement at the interface in the cohesive zone, which is known as the traction–separation law or cohesive law, is used to describe the adhesion between materials. The form of the traction–separation law, such as the commonly encountered Dugdale law, bilinear law, and exponential law, as shown in Figure 4, is crucial to the effectiveness of simulating the interface [25]. The essence of the traction–separation law is to characterize the interaction between atoms or molecules of the material [26]. For the microtransfer printing technique, it is usually carried out in dry and uncharged environments, and the van der Waals force is the main source of the interaction between atoms or molecules. Therefore, the normal interaction at the stamp/film interface can be characterized by the Lennard–Jones surface force law derived from the intermolecular pair potential, which is written as [27]

(3) $$T_{n}=\frac{8\Delta \gamma }{3\varepsilon }\left[{\left(\frac{\varepsilon }{\Delta_{n}+\varepsilon }\right)}^{3}-{\left(\frac{\varepsilon }{\Delta_{n}+\varepsilon }\right)}^{9}\right]$$

where *T*_n_ is the normal adhesive force per unit area between two surfaces, Δ_n_ denotes the surface relative displacement, Δ*γ* is the work of adhesion, and *ε* denotes the equilibrium distance between two flat surfaces.

Comparisons between the Lennard–Jones surface force law and three commonly used traction–separation laws are depicted in Figure 4, in which *T*_max_ denotes the maximum traction force and *δ*_n_ is the characteristic length in the normal direction. It can be seen that the traction–separation displacement relationship described by the exponential law is close to that described by the Lennard–Jones surface force law, which can be used to analyze the adhesion/delamination problem at the stamp/film interface.

The control equations of the exponential law in the two-dimensional plane state [28] are

(4) $$T_{n}=-\frac{\phi_{n}}{\delta_{n}}\exp\left(-\frac{\Delta_{n}}{\delta_{n}}\right)\left\{\frac{\Delta_{n}}{\delta_{n}}\exp\left(-\frac{\Delta_{t}^{2}}{\delta_{t}^{2}}\right)+\frac{1-q}{r-1}\left[1-\exp\left(-\frac{\Delta_{t}^{2}}{\delta_{t}^{2}}\right)\right]\left(r-\frac{\Delta_{n}}{\delta_{n}}\right)\right\}$$

(5) $$T_{t}=-\frac{\phi_{n}}{\delta_{n}}\left(2\frac{\delta_{n}}{\delta_{t}}\right)\frac{\Delta_{t}}{\delta_{t}}\left\{q+\left(\frac{r-q}{r-1}\right)\frac{\Delta_{n}}{\delta_{n}}\right\}\exp\left(-\frac{\Delta_{n}}{\delta_{n}}\right)\exp\left(-\frac{\Delta_{t}^{2}}{\delta_{t}^{2}}\right)$$

(6) $$\phi_{n}=e\cdot \sigma_{\max}\cdot \delta_{n}$$

where *T*_n_ and *T*_t_ are normal and tangential tractions across the surface, respectively. *ϕ*_n_ is the fracture energy of normal separation. *σ*_max_ is the normal strength at the cohesive surface, that is, the maximum stress. Δ_n_ and Δ_t_ are the interface separation displacements in the normal and tangential directions, respectively, and *δ*_n_ and *δ*_t_ are the corresponding characteristic lengths. *q* = *ϕ*_t_/*ϕ*_n_ and *r* = Δ_n_*/*δ*_n_ are the coupling constants between the normal and tangential directions. *ϕ*_t_ is the fracture energy of tangential separation. Δ_n_* is the normal displacement after complete shear separation under *T*_n_ = 0.

**Figure 5:**
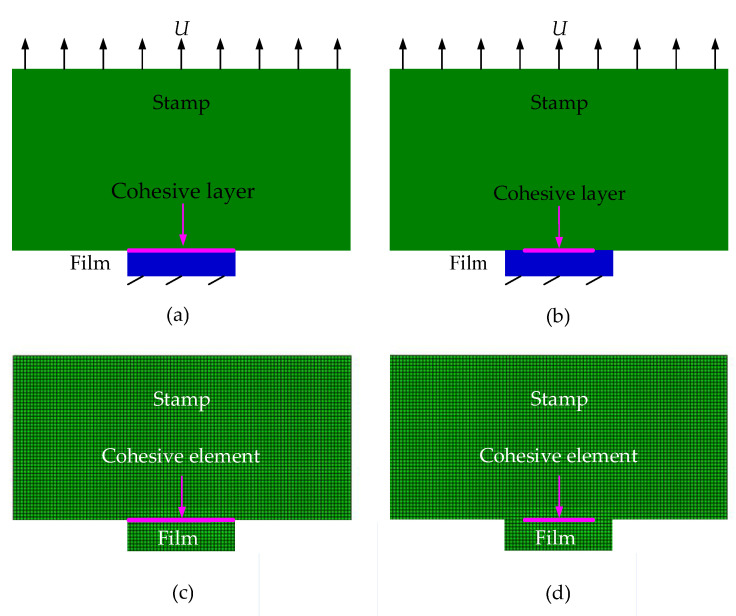
Schematics of models based on the CZM for the stamp/film interface. (**a**) Mechanical model without preset cracks; (**b**) Mechanical model with preset cracks; (**c**) Finite element model without preset cracks; (**d**) Finite element model with preset cracks.

In order to analyze the interface adhesion/delamination problem by combining the CZM with the finite element method, cohesive elements with properties following the traction–separation law should be preset along the interface, as shown in Figure 5a. In addition, the model shown in Figure 5b, which introduces initial cracks with length *c* at both ends of the interface through inserting cohesive elements only in the middle region of the stamp/film interface, is also established. This model is used to compare with the model based on the VCCT and investigate the influence of initial cracks on the mechanical behaviors of the interface. The two-dimensional finite element models established in Abaqus are shown in Figure 5c,d. Since the exponential law represented by Equations (4) and (5) is not available as cohesive elements in commercial finite element software, the user subroutine approach [29] is used in the present analysis to develop user-defined cohesive zone elements at the stamp/film interface.

In the above three kinds of finite element models, the stamp and film are simulated by the plane strain reduction integral element (CPE8R), and the number of elements and nodes are determined by the mesh-independent analysis. The boundary conditions are that the bottom boundary of the film is constrained and the displacement load is applied on the top boundary of the stamp.

## 3. Results and Discussions

### 3.1. Validation of the Modeling Methods

**Figure 6:**
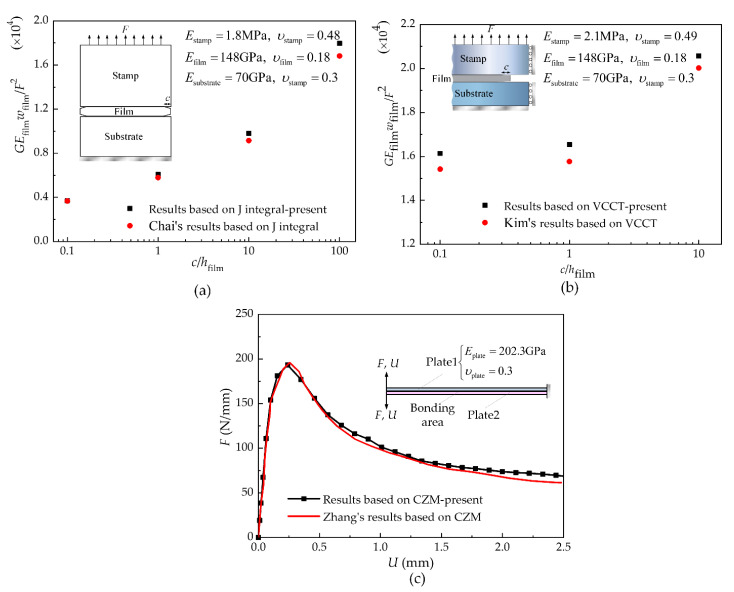
Comparisons between the results of the present models based on J-integral theory, the VCCT, and the CZM and those of references. (**a**) Comparisons between results of the present model based on J-integral theory and those of reference [30]; (**b**) Comparisons between results of the present model based on VCCT and those of reference [11]; (**c**) Comparisons between results of the present model based on CZM and those of reference [31].

In order to verify the finite element modeling methods based on J-integral theory, the VCCT, and the CZM presented in Section 2, this paper compares the results with those of Chai et al. [30], Kimlee et al. [11], and Zhang et al. [31] through establishing the same models as them, as shown in Figure 6. Figure 6a,b demonstrates the results of the models based on J-integral theory and the VCCT, respectively. In these models, the stamp, film and substrate are assumed to be elastic materials with Young’s moduli of *E*_stamp_, *E*_film_, and *E*_substrate_ and Possion’s ratios of *υ*_stamp_, *υ*_film_, and *υ*_substrate_, respectively. The width and thickness of the film are *w*_film_ and *h*_film_. A crack of length *c* is preset at the stamp/film interface, and a tensile force *F* is applied at the top of the stamp. It can be seen that the dimensionless energy release rate at the stamp/film interface obtained by these two models increases with the increase in the dimensionless initial crack length. Furthermore, the present results are similar to those in references [11,30], and discrepancies between them may be caused by the difference in mesh sizes. Figure 6c depicts results of the model based on the CZM for the thin-plate bonding structure model in which the same structure size and parameters of the CZM as with Zhang’s case [31] are set in the present analysis. Although the present paper adopts the user-defined element subroutine (UEL) approach to develop cohesive elements, while reference [31] adopts the user-defined material subroutine (VUMT) approach, their results show good agreements. The above comparisons illustrate the correctness of the present modeling methods.

### 3.2. Comparisons between the Results of Different Mechanical Models and Experiments

This section intends to carry out finite element simulations based on the three mechanical models for the stamp/film interface established in Section 2, and compares the prediction results with experimental ones in Kim’s study [32]. The analysis condition in the simulations is set to be in agreement with the experimental case [32], in which the width and thickness of the silicon film are 100 μm and 3 μm, respectively, and its Young’s modulus and Poisson’s ratio are 130 GPa and 0.18. For the viscoelastic stamp used in the microtransfer printing, its material is simplified to be elastic in this section so that comparisons of results between the three models can be conducted and because the relationship between the energy release rate and the J-integral for a viscoelastic interface is still unclear. The Young’s modulus and Poisson’s ratio used for the stamp are 1.8 MPa and 0.49 [32], respectively. Relevant parameters in the traction–separation law are shown in Table 1.

**Figure 7:**
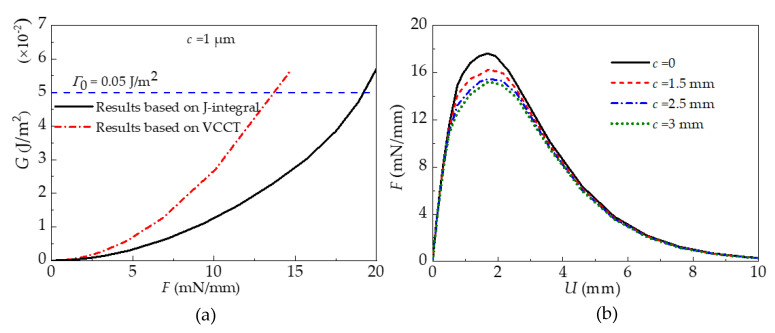
Variations in the energy release rate or normal force calculated by the models based on the J-integral, VCCT, and CZM. (**a**) Variations of energy release rate G with normal force F for the models based on J-integral and VCCT; (**b**) Variations of normal force F with displacement U for the model based on CZM.

**Figure 8:**
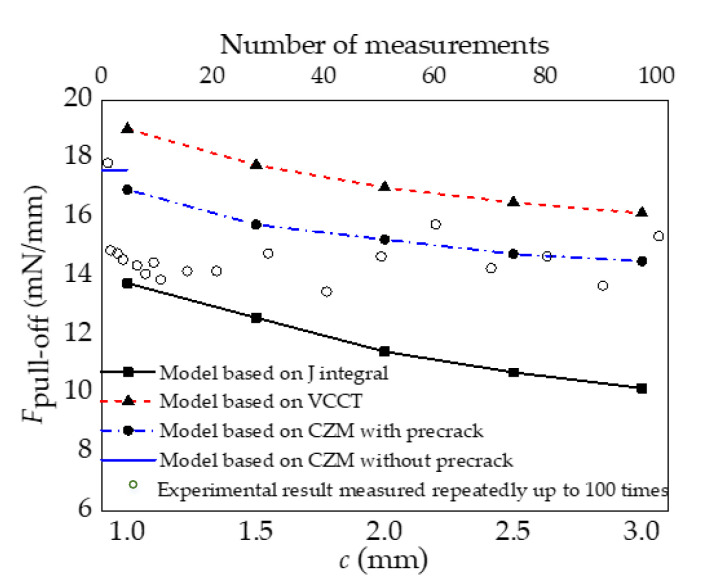
Variations in the pull-off force *F*_pull-off_ with a preset crack length *c* calculated by three models and those measured repeatedly in the experiments of reference [32].

Variations in the energy release rate or normal force calculated by the models based on J-integral theory, the VCCT, or the CZM are shown in the Figure 7. It is found from Figure 7a that the energy release rate obtained by both the model based on J-integral theory and that based on the VCCT increases with the increase in the normal force under the same preset crack size. However, the magnitude of the energy release rate is different. According to the Griffith criterion, the interface begins to delaminate when the energy release rate *G* reaches the fracture toughness *Γ*_0_. The normal force at this critical state is defined as the pull-off force, which is used to characterize the strength of interfacial adhesion. For the PDMS stamp/silicon film interface, the typical value of the fracture toughness is *Γ*_0_ = 0.05 J/m^2^ [15]. It can be seen from Figure 7b that the normal force calculated by the model based on the CZM for both the interface with a preset crack and that without a preset crack increases with the increase in displacement until it reaches the maximum value. At this juncture, the crack is generated and the interface reaches the critical state of delamination. The force in this state is defined as the pull-off force in this model. After the juncture, the normal force decreases with the increase in displacement due to the propagation of cracks. The pull-off force results extracted from these three models are depicted in Figure 8. As can be seen, the pull-off force decreases with the increase in the preset crack length, which demonstrates the dependence of interface delamination on the initial crack. In addition, the pull-off force predicted by the model based on the CZM falls in between the results predicted by the model based on J-integral theory and that based on the VCCT. The pull-off force between a flat stamp and a silicon platelet measured by Kim et al. [32] are also presented in Figure 8 for comparison. These experimental data are obtained by repeated measurements up to 100 times, which can reflect the influences of the crack length to some extent as the initial flaw in every measurement may be different. Through dividing the total measured pull-off force by the length of the silicon platelet, the experimental results can be compared with the simulation ones, as the simulations are conducted under the plane strain condition. After comparison, it is found that the simulation results of the model based on the CZM are closer to the experimental data than the model based on the J-integral and that based on the VCCT.

In summary, initial cracks need to be preset in both the model based on J-integral theory and that based on the VCCT, and the delamination behaviors of the film/stamp interface predicted by these two models change with the preset crack length. However, in practical microtransfer printing processes, the initial crack size at the stamp/film interface is unpredictable [11,15], which makes these methods have limitations. For the model based on the CZM, the results show better agreement with the experimental data, which reflects its feasibility in predicting the interface delamination. Furthermore, this model has the ability to predict the initiation and propagation of interface delamination without presetting initial cracks, and, therefore, it is more suitable for analyzing the adhesion/delamination behavior of the stamp/film interface compared with the models based on J-integral theory and the VCCT.

### 3.3. Analysis of the Mechanical Properties at the Stamp/Film Interface

The studies in Section 3.2 exhibit the suitability of the model based on the CZM in analyzing the mechanical behaviors of the stamp/film interface under the assumption of an elastic stamp. However, the widely used PDMS stamp demonstrates intrinsic time-dependent viscoelastic properties. Neglect of the viscous dissipation which may occur at the stamp/film interface during retracting is unreasonable. This section intends to investigate the adhesion/delamination mechanical behaviors at the viscoelastic stamp/film interface using the model based on the CZM without presetting initial cracks and analyze the effects of the pulling speed and interfacial parameters on the pull-off force. For the PDMS stamp under investigation, its hyperelastic properties are described by the Mooney-Rivlin strain energy function with parameters *C*_10_ = 0.243243 MPa, *C*_01_ = 0.0608108 MPa, and *D*_1_ = 0.131556 MPa [23], and its viscoelastic properties are described by the generalized Maxwell model in Prony series with parameters *g*_1_ = 0.391892 and *τ*_1_ = 0.08 [23]. The width and thickness of the silicon film are set to be 50 μm and 5 μm, respectively. The same constitutive parameters in the CZM for the stamp/film interface as those in Section 3.2 are adopted.

#### 3.3.1. The Effect of Pulling Speed

**Figure 9:**
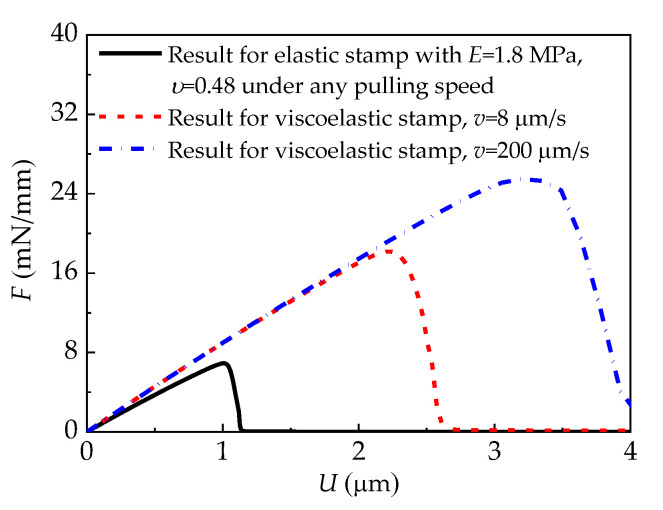
Comparisons of the force–displacement curves for the elastic stamp and the viscoelastic stamp.

**Figure 10:**
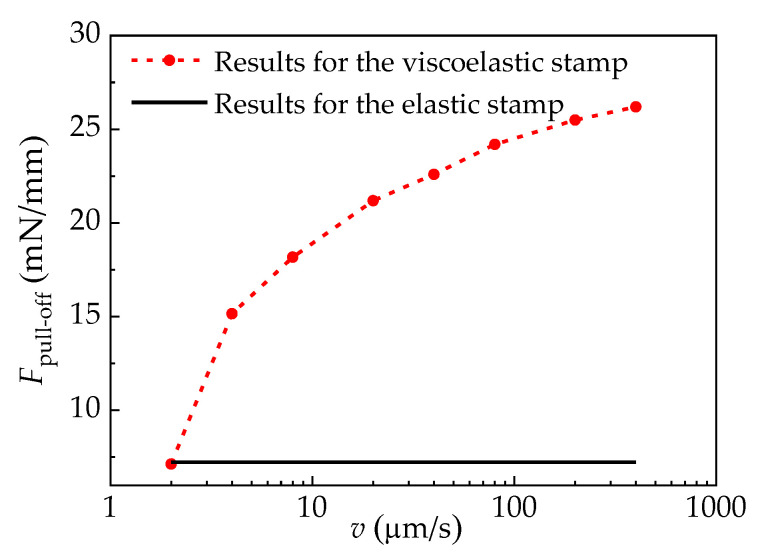
Effects of pulling speed on pull-off force.

The influences of the viscoelastic properties of the stamp on the force–displacement curves are shown in Figure 9, in which the Young’s modulus and Poisson’s ratio of the elastic stamp are set to be equal to the corresponding initial parameters of the viscoelastic stamp. It is found that the force–displacement curve is independent of the pulling speed of the elastic stamp, while it is dependent on the pulling speed of the viscoelastic stamp. Furthermore, to illustrate the influence of viscoelasticity on the interface adhesion strength, Figure 10 depicts variations of the pull-off force under different pulling speeds. As can be seen, the pull-off force for the viscoelastic stamp/film interface increases with the pulling speed, reflecting the stamp’s viscous response effect. This result implies that delamination between the viscoelastic stamp and the film is more likely to occur at a lower pulling speed.

#### 3.3.2. The Effect of Interfacial Cohesive Constitutive Parameters

**Figure 11:**
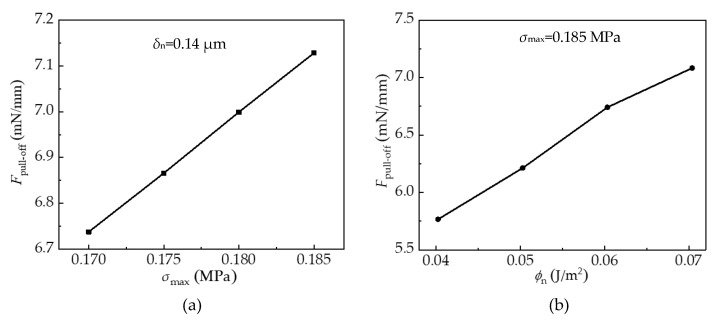
Effects of the cohesive constitutive parameters on the pull-off force. (**a**) Effects of normal strength; (**b**) Effects of normal fracture energy.

Although the cohesive zone model using the exponential law described in Section 2.3 includes the fracture mode mixity, the dominant mode in stamp/film delamination during retraction of the stamp is the opening mode. Therefore, the results presented in this section are discussed in terms of the dominant normal strength *σ*_max_ and the normal fracture energy *ϕ*_n_. Figure 11a,b present variations of the pull-off force with the normal strength and the normal fracture energy at the viscoelastic stamp/film interface under the pulling speed of 2 μm/s. It is found that the pull-off force increases almost linearly with the increasing normal strength under a fixed characteristic length *δ*_n_. This implies that a larger normal strength can enhance the adhesion between stamp and film, which is beneficial to the success of the picking-up process in microtransfer printing. Furthermore, increases in the normal fracture energy can also increase the pull-off force when the normal strength is fixed. According to the relationship *ϕ*_n_ = *eσ*_max_*δ*_n_ [28], the normal fracture energy is proportional to the normal characteristic length *δ*_n_ which denotes the normal interfacial separation for damage initiation. Therefore, the larger the normal fracture energy, the later the damage initiation, and the more difficult the interface delamination.

## 4. Conclusions

Mechanical models based on J-integral theory, the VCCT, and the CZM for predicting adhesion/delamination behaviors at the stamp/film interface were developed and simulated through finite element modeling in this study. The pull-off force required to fail the interface were extracted from the results of these three models, and its variations with the preset crack length were presented. Through comparing between the simulation results and previous experimental results, the choice of a priority mechanical model was determined. Furthermore, to provide insight into the mechanical characteristics at the stamp/film interface, the effects of the microtransfer printing technological parameter and interface material parameters on the pull-off force were inspected based on the priority model. The main conclusions are as follows.

The major disadvantage for models based on J-integral theory and the VCCT was the introduction of initial fictitious cracks which made the delamination behaviors of the stamp/film interface change with the preset crack length. The model based on the CZM could not only predict results that were close to the model based on the VCCT in the presence of fictitious cracks, but also predict the initiation and propagation of interface delamination without presetting initial cracks. Furthermore, the simulation results of the model based on the CZM are closer to the previous experimental data, exhibiting its suitability in analyzing the adhesion/delamination behavior of the stamp/film interface.

Simulation results of the model based on the CZM without presetting initial cracks indicated that the stamp/film interface adhesion strength characterized by the pull-off force was independent of the pulling speed under the elastic assumption of the stamp, while it increased with the pulling speed when accounting for the viscoelastic properties of the stamp. Furthermore, the pull-off force of the viscoelastic stamp/film interface tended to increase with increases in the normal strength and the normal fracture energy, which was beneficial to the success of the picking-up process in microtransfer printing.

## Figures and Tables

**Table 1:** Constitutive parameters in the CZM for the stamp/film interface.

*σ*_max_ (MPa)	*δ*_n_ (μm)	*δ*_t_ (μm)	*q*	*r*
0.185	0.14	0.12	1	0

## Data Availability

Not applicable.
